# Protective Effects of a *Brassica nigra* Sprout Hydroalcoholic Extract on Lipid Homeostasis, Hepatotoxicity, and Nephrotoxicity in Cyclophosphamide-Induced Toxicity in Rats

**DOI:** 10.3390/metabo14120690

**Published:** 2024-12-08

**Authors:** Hassan Barakat, Thamer Aljutaily, Raghad I. Alkhurayji, Huda Aljumayi, Khalid S. Alhejji, Sami O. Almutairi

**Affiliations:** 1 Department of Food Science and Human Nutrition, College of Agriculture and Food, Qassim University, Buraydah 51452, Saudi Arabia; thamer.aljutaily@qu.edu.sa (T.A.); 441111971@qu.edu.sa (K.S.A.); 2 Al Bukayriyah General Hospital, Prince Niaf Ibn Abdulaziz, Ar Rawdah, Qassim Health Cluster, Ministry of Health, Al Bukayriyah 52725, Saudi Arabia; rialkhurayji@moh.gov.sa; 3 Department of Food Science and Nutrition, College of Sciences, Taif University, P.O. Box 11099, Taif 21944, Saudi Arabia; huda.a@tu.edu.sa; 4 Dariyah General Hospital, King Khalid Road, Qassim Health Cluster, Ministry of Health, Dariyah 58523, Saudi Arabia; 5 Department of Clinical Nutrition, Almethnab General Hospital, Qassim Health Cluster, Ministry of Health, Al Mithnab 56526, Saudi Arabia; soalmutairi@moh.gov.sa

**Keywords:** *Brassica nigra*, polyphenols, liver and kidney function, antioxidant, hyperlipidemia, food supply

## Abstract

**Background:** *Brassica nigra* possesses a significant concentration of bioactive compounds and has been demonstrated to have a variety of pharmacological properties, although its sprout has not been extensively studied. Thus, the protective effects of *Brassica nigra* sprout hydroalcoholic extract (BNSE) on lipid homeostasis, hepatotoxicity, and nephrotoxicity in cyclophosphamide (CYP)-induced toxicity in rats were examined in this study. **Methods:** Four experimental rat groups (*n* = 8 for each group) were examined as follows: NR, normal rats that received normal saline by oral gavage daily; CYP, injected with a single dose of CYP at 250 mg kg^−1^ intraperitoneally (i.p.) and did not receive any treatment, receiving only normal saline by oral gavage daily; CYP + BNSE250, injected with a single dose of CYP at 250 mg kg^−1^ i.p. and treated with BNSE at 250 mg kg^−1^ by oral gavage daily for three weeks; and CYP + BNSE500, injected with a single dose of CYP at 250 mg kg^−1^ i.p. and treated with BNSE at 500 mg kg^−1^ by oral gavage daily for three weeks. **Results:** The results indicated a significant increase (*p* < 0.05) in triglyceride (TG), cholesterol (CHO), low-density lipoprotein cholesterol (LDL-c), and very low-density lipoprotein cholesterol (VLDL-c) levels in CYP-induced toxicity rats. The administration of BNSE at 250 and 500 mg kg^−1^ significantly (*p* < 0.05) attenuated TG, CHO, LDL-c, and VLDL-c at values comparable with the NR group. The most efficient treatment for improving the lipid profile and atherogenicity complication was BNSE at 500 mg kg^−1^, performing even better than 250 mg kg^−1^. Administrating BNSE at 250 or 500 mg kg^−1^ improved the liver’s function in a dose-dependent manner. Comparing the lower dose of 250 mg kg^−1^ of BNSE with 500 mg kg^−1^ showed that administrating 250 mg kg^−1^ attenuated alanine transaminase (ALT) by 28.92%, against 33.36% when 500 mg kg^−1^ was given. A similar trend was observed in aspartate aminotransferase (AST), where 19.44% was recorded for BNSE at 250 mg kg^−1^ and 34.93% for BNSE at 500 mg kg^−1^. Higher efficiency was noticed for BNSE at 250 and 500 mg kg^−1^ regarding alkaline phosphatase (ALP). An improvement of 38.73% for BNSE at 500 mg kg^−1^ was shown. The best treatment was BNSE at 500 mg kg^−1^, as it markedly improved liver function, such as total bilirubin (T.B.), in a dose-dependent manner. The administration of BNSE attenuated the total protein (T.P.), albumin, and globulin levels to be close to or higher than the typical values in NR rats. **Conclusions:** BNSE might be used for its promising hypolipidemic, hepatoprotective, and nephroprotective potential and to prevent diseases related to oxidative stress. Further research on its application in humans is highly recommended.

## 1. Introduction

Eating more plant-based meals has many health benefits, including better nutrition, greater resilience to disease, and better protection against oxidative stress. Since traditional pharmaceuticals are prohibitively expensive, the World Health Organization (WHO) estimates that as many as 80% of people in developing nations rely on locally available plant resources for their medical needs [1]. For example, the abundant secondary metabolites produced by brassica plants have several positive biological effects [2]. These include a variety of micronutrients, such as phenols, polyphenols, flavonoids, carotenoids, alkaloids, phytosterols, chlorophyll, glucosinolates, terpenoids, and glucosides [3]. Tocopherols, terpenes, phytoalexins, and phytosterols are also present [2,4]. Their abundance of minerals, such as calcium, magnesium, copper, iron, selenium, and zinc, aids in the body’s ability to recover from illnesses, resist diseases, and boost immunity [5]. Avato and Argentieri [6] claimed that glucosinolates (GSLs) and tocopherols are among the nutrients found in mustard seeds. These GSLs are most commonly found in aliphatic, aromatic, or indolic forms [7] and possess properties that mitigate the effects of cancer and oxidative stress [8]. Aliphatic GSLs, such as synegrins, are common and have many valuable properties, including anticancer, antibacterial, antifungal, antioxidant, anti-inflammatory, wound healing, and biofouling mechanisms [9]. In addition to their anticancer and antibacterial properties, allyl isothiocyanate and sulforaphane have anticancer, antibacterial [10], antifungal [11], and anti-inflammatory properties [5].

*B. nigra*, as one of the brassica plants, has antidiabetic characteristics [12] and anticonvulsant properties [13], promotes and improves blood flow [14], reduces the risk of cardiovascular illness [15,16], and activates antioxidant enzymes, which in turn promotes immune cells [17], while simultaneously exhibiting antiproliferative [18] and antibacterial properties [19]. Sprouts have many health benefits, including their abundance of antioxidant-rich phenolics and non-phenolics, which have only recently been revealed [20], as well as GSL capacities to engage in ultra-extreme scavenging activity [21]. Thus, nutrients are more concentrated in immature, sprouted seeds and greens than in older, ripe seeds. Researchers and practitioners from all over the globe have started paying attention to this plant as its popularity as a functional food has grown [22]. Regardless of their nutritional importance, *B. nigra* seeds are rarely examined. Because of its high nutrient content, sprout consumption has risen in Western Europe [23]. The high levels of protein, polyunsaturated fatty acids, vitamins, and minerals in the sprout give the impression that it is more nutritious than the seed [23]. Sprouted meals facilitate the digestion of proteins, carbohydrates, and lipids by activating enzymes [23]. *B. nigra* sprouts have cancer prevention and [24] treatment activity [25] in several organs. They are of interest to those who are looking to limit sugar accumulation because they can either block or delay the digestion of carbohydrates by employing enzymes like α-glucosidase [26], inhibiting endothelial cell inflammatory responses and hence decreasing the generation of advanced glycation end products [27], as well as enhancing insulin resistance [28].

Conversely, the importance of sprouts from related seeds in heart-related illnesses has been confirmed by in vitro and in vivo research [29]; they reduce cholesterol levels in the liver [30], improve cholesterol metabolism while decreasing signs of oxidative stress [31], alleviate long-term inflammation [32], decrease the prevalence of viruses [33], and possess stress-relieving and anti-atherosclerotic properties [34]. Recent research in rats has demonstrated that *B. nigra* spouts can enhance the immune system’s ability to fight off CYP-induced immunosuppression [35] and exhibit nephroprotection efficiency [36]; however, research on their nephroprotective and hepatoprotective effects is still in its early stages. Research has shown that *B. nigra* has a wide variety of pharmacological properties, including antioxidant, anti-inflammatory, antiepileptic, and antidiabetic properties. *Brassica rapa* also has hypolipidemic, immunological, anti-inflammatory, anti-obesity, antidiabetic, hepatoprotective, nephroprotective, lung-protective, and mutagenic [37] effects, among others [11,37]. However, to the best of our knowledge, this study is the first to investigate the hepatoprotective and nephroprotective activity of *B. nigra* sprouts in CYP-induced toxicity in rats. A better understanding of brassica sprouts’ nutritional, therapeutical, and versatile benefits may make them more significant in supporting health. BNSE sprouts may improve our health and support a more sustainable food system.

Consequently, the present study set out to examine whether or not a hydroalcoholic extract of *B. nigra* had any nephroprotective or hepatoprotective effects in rats. Achieving this required the preparation of *B. nigra* sprouts in a controlled environment and the monitoring of bioactive chemicals throughout the sprouting process. Next, biomarkers for the liver and kidneys were used to evaluate the biological efficacy of a hydroalcoholic extract produced from the finest *B. nigra* sprouts.

## 2. Materials and Methods

### 2.1. Raw B. nigra Seeds

The Brassica seeds (*Brassica nigra* L.) were bought from True Leaf Market, an online garden center (www.trueleafmarket.com) accessed on 22 February 2023. Until needed, two kilos of unadulterated seeds were preserved in a dry and cool place.

### 2.2. B. nigra Sample Preparation and Sprouting

Before examination, the raw mustard seeds were passed through stainless-steel sieves to eliminate dust or extraneous elements. Following a 2 min soak in a 2% sodium hypochlorite solution, the seeds were superficially cleaned and then sprouted in 50 g batches following the protocol of Barakat et al. [38]. The seed germination process was conducted in a climate-controlled environment with a relative humidity of 90% and a temperature of 17 ± 1 °C, using an atomizer from EasyGreen, model EGL 50, Elvington, UK. The sprouts of *B. nigra* were quickly frozen at −18 ± 1 °C and then subjected to lyophilization for 96 h at −48 °C (CHRISTMALPHA 1-2 LD plus, Osterode am Harz, Germany) and 0.032 mbar. Secondly, to conduct the in vivo biological evaluation of the *B. nigra* sprouts, 2 kg of stalks were sprouted independently under the same conditions for 6 days. They were then dried gradually according to a 24 h drying program, crushed, sieved, and extracted [38]. The *B. nigra* sprout hydroalcoholic extract (BNSE) was prepared by extracting around 500 g of sprouts three times with 2500 mL of 50% ethanol. The filtered extract was concentrated using a rotary evaporator while the solvent was evaporated at 40 °C. Then, it was freeze-dried for 96 h at −52 °C and 0.032 mbar [39]. The freeze-dried extract was dissolved in a normal saline solution for rat treatment, and the doses were calculated depending on the rat’s body weight, as mentioned below.

### 2.3. Rat Experiment Design

In this study, 32 adult male Wistar albino rats weighing 180–200 g and aged 6–8 weeks were used. This research was approved by Qassim University’s Institutional Animal Ethics Committee (IAEC) (Approval No. 23-19-08, Monday, 5 January 2023). For rat adaptation, the animals were housed in air-conditioned polypropylene cages at 24 ± 1 °C for one week, with free access to commercial feed and water *ad libitum* under a 12 h light/darkness cycle. Four groups, with eight rats in each, were examined in the experiment. G1 comprised normal rats (NR) that received normal saline by oral gavage daily. A dose of 250 mg CYP kg^−1^ body weight (bw) was freshly prepared and injected intraperitoneally (i.p.) in groups G2, G3, and G4 [35]. G2: CYP, injected with CYP and did not receive any treatment, only receiving normal saline by oral gavage daily; CYP + BNSE250, injected with CYP and administrated 250 mg kg^−1^ bw by oral gavage daily; and CYP + BNSE500, injected with CYP and administrated 500 mg kg^−1^ bw by oral gavage daily for three weeks. The body weight was measured for each rat weekly for relative weight gain calculation. For the various biochemical examinations, the rats were anesthetized (intramuscular injection of ketamine/xylazine hydrochloride mix (80/10 *v:v*)) at the end of 3 weeks. At 24 h after the last dose of BNSE, blood samples were collected, and the serum was separated by centrifugation for 30 min at 4000 rpm under 10 °C.

#### 2.3.1. Estimation of the Relative Weight of the Liver, Kidneys, and Spleen

Three rats from each group were cervically dislocated after anesthetizing and blood sampling. Before computing the relative weight, the liver, kidneys, and spleen were carefully removed and weighed on a precise scale.

#### 2.3.2. Determination of Lipid Profile and Atherogenic Index

The lipid profile, including triglycerides (TG, mg dL^−1^) and total cholesterol (TC, mg dL^−1^), was examined using an enzymatic colorimetric test kit based on the GPO-PAP method [40]. High-density lipoprotein cholesterol (HDL-c, mg dL^−1^) was quantified with an enzymatic colorimetric direct homogeneous assay kit in accordance with the manufacturer’s instructions [41]. Low-density lipoprotein cholesterol (LDL-c, mg dL^−1^) and very-low-density lipoprotein cholesterol (VLDL-c, mg dL^−1^) were computed numerically in accordance with Friedewald et al. [42]. The atherogenic index (AI), an essential predictor of atherosclerosis that reflects the ratio of TG and HDL-c [43], was calculated following the formula Log_10_ (TG/HDL-c).

#### 2.3.3. Determination of Liver and Kidney Function

The liver’s function was assessed by measuring the alanine aminotransferase (ALT, UL^−1^), aspartate aminotransferase (AST, UL^−1^), alkaline phosphatase (ALP, UL^−1^), and total bilirubin (T.B., mg dL^−1^) in the blood serum using specific kits: an alanine aminotransferase kit (EC 2.6.1.2), aspartate aminotransferase kit (EC 2.6.1.1), optimum alkaline kit (EC 3.1.3.1), and photometric test kits for total bilirubin. The concentrations of total protein (T.P., g dL^−1^), albumin (g dL^−1^), creatinine (mg dL^−1^), and urea (mg dL^−1^) were measured using photometric and colorimetric test kits, employing the Biuret method for total protein, the BCG method for albumin, and a fully enzymatic test kit utilizing the GLDH method, following the manufacturer’s instructions. Globulin (g dL^−1^) was determined by subtracting albumin from the total protein quantities. Blood urea nitrogen (BUN, mg dL^−1^) was determined by multiplying the urea concentration by 0.47. All biochemical testing kits were acquired from Human Co., Wiesbaden, Germany. All kits were transported under cooling conditions and handled following the manufacturer’s instructions.

### 2.4. Statistical Analysis

Statistical analysis was conducted using SPSS (Version 22.0 for Windows, IBM, Houston, TX, USA). Experimental findings were presented as the mean ± SE. Statistical significance was assessed using one-way ANOVA followed by a post hoc test, with *p*-values < 0.05, as per Steel et al. [44].

## 3. Results and Discussion

### 3.1. Effect of B. nigra Sprout Extracts on Weight Gain % and Organs’ Relative Weight

The percentage of weight increase and the relative organ weight in rats with CYP-induced immunosuppression are shown in Table 1. The rats’ weights decreased directly after the CYP injection in the first week, but the treated groups’ weights decreased less toward the end of the experiment. The rats’ weights were best restored by 500 mg kg^−1^ BNSE. Low-dose BNSE was the least effective weight gain booster compared to NR. Weight recovery was associated dose-dependently with the BNSE content. Table 1 shows the immunosuppressive effects of CYP, a clinical chemotherapeutic drug, including weight loss and organ weight decreases. Liver and nephrotoxicity may ensue from its cytotoxicity [45,46,47].

Notable enhancements were observed in the relative organ weight percentages of the kidneys, liver, and spleen following the administration of BNSE at dosages of 250 and 500 mg kg^−1^, particularly at 500 mg kg^−1^. The weights of the liver and kidneys exhibited considerable variation among all groups. The BNSE group at 500 mg kg^−1^ exhibited the most significant liver weight, whereas the CPY-treated rat group displayed the lowest liver weight, with a statistically significant difference (*p* < 0.05). Likewise, the kidney and spleen weights were influenced by CYP injection. A substantial disparity in their weights and a high dosage of BNSE were noted. Evidence suggests that brassica plants can positively affect weight gain in rats through various mechanisms, such as bioactive compounds’ presence and gut microbiota modulation [48,49]. These findings highlight the potential of brassica vegetables as a beneficial component of the diet, aimed at promoting healthy weight gain and overall metabolic health. Further research is needed to explore the implications of these findings for human nutrition and dietary recommendations.

### 3.2. The Hypolipidemic Effects of B. nigra Sprout Extract

Table 2 reveals that 250 and 500 mg kg^−1^ BNSE had hypolipidemic effects in rats with CYP-induced immunosuppression. TG, CHO, LDL-c, and VLDL-c increased significantly (*p* < 0.05) in immunosuppressed rats treated with CYP. The CYP injection drastically lowered the HDL levels in rats. BNSE at 250 and 500 mg kg^−1^ bw significantly attenuated the TG, CHO, LDL-c, and VLDL-c levels compared to the NR and CYP groups. The blood profile improved more significantly with 500 mg kg^−1^ BNSE than with 250 mg kg^−1^. At 250 and 500 mg kg^−1^, BNSE reduced TG by 34.92 and 46.10% in rats. HDL-c rose by 27.15 and 38.84% with 250 and 500 mg kg^−1^ BNSE. BNSE at 250 and 500 kg^−1^ lowered LDL-c to 65.65 and 65.38%. Dose-dependent VLDL-c improved. BNSE 500 mg kg^−1^ reduced VLDL-c by 46.10% more than CYP.

BNSE dramatically reduced and improved the total glycerides, cholesterol, and its derivatives. These results can be interpreted based on the phytochemicals found in BNSE, as analyzed in another part of this study [35]. It may operate as a hyperlipidemic and hypercholesterolemic agent, which is entirely consistent with Lee et al. [50], who employed a *B. juncea* leaf extract to examine fat accumulation and lipid profiles. The current evidence suggests that *B. nigra* sprout extract may be used to treat dyslipidemia; however, the mechanisms remain to be studied.

### 3.3. Effect of B. nigra Sprout Extract on the Atherogenic Index

**Figure 1:**
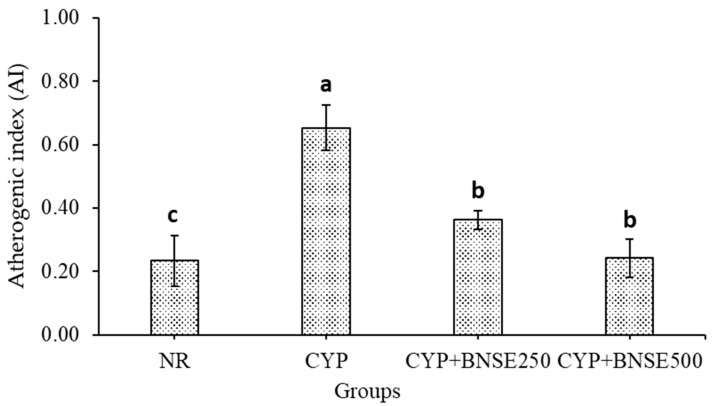
Effects of *B. nigra* sprout extract at different doses on AI in rats with CYP-induced immunosuppression (mean ± SE), *n* = 8. ^a,b,^ and ^c^: bars not sharing similar letters differed significantly (*p* > 0.05), for experimental groups, see Materials and Methods.

The efficacy of BNSE at 250 and 500 mg kg^−1^ on the AI in rats with CYP-induced immunosuppression was assessed; the results are depicted in Figure 1. The AI was markedly elevated following CYP injection in the CYP group compared to the NR group. The most effective treatment in reducing atherogenicity complications was BNSE at 500 mg kg^−1^ CYP + BNSE500, which showed greater efficacy than when applying BNSE at 250 mg kg^−1^ (CYP + BNSE250). Lee et al. [50] suggested that the phytochemicals in BNSE demonstrate hyperlipidemic and hypercholesterolemic effects on fat accumulation and lipid profiles. Consequently, the AI may be enhanced as *B. nigra* sprout extract demonstrates clinical efficacy in the treatment of dyslipidemia.

### 3.4. Effects of B. nigra Sprout Extract on Liver Function

Table 3 shows how the BN extract at 250 and 500 mg kg^−1^ affected the liver function of CYP-induced immunosuppressed rats. In G2 rats, CYP injection significantly (*p* < 0.05) increased the serum ALT, AST, and ALP enzyme levels compared to the NR group. The T.B. levels increased considerably in CYP-treated rats (Table 3). The administration of BNSE at 250 or 500 mg kg^−1^ improved the liver function in a dose-dependent manner. A higher dose of BNSE was more effective than a smaller dose in restoring liver function changes caused by CYP injection to near-normal levels in NR (Table 3). Comparing the lower dose of 250 mg kg^−1^ of BNSE to 500 mg kg^−1^ revealed that administering 250 mg kg^−1^ reduced ALT by 28.92%, versus 33.36 when 500 mg kg^−1^ was given. A similar pattern was seen in AST, with 19.44% found for BNSE at 250 mg kg^−1^ and 34.93% for BNSE at 500 mg kg^−1^. Regarding ALP, BNSE at 250 and 500 mg kg^−1^ showed higher efficiency. A 38.73% improvement was observed with BNSE at 500 mg kg^−1^. The best treatment was BNSE at 500 mg kg^−1^, which significantly (*p* < 0.05) improved the liver enzymes (as measured by ALT, AST, and ALP) and several liver functions, such as T.B., in a dose-dependent manner. The T.P., albumin, and globulin levels were considerably lower in CYP-treated rats (Table 3). High dosages of BNSE significantly (*p* < 0.05) boosted the T.P., albumin, and globulin levels in BNSE-treated rats, bringing them close to or beyond the usual values (Table 3). Compared to the NR group, the most efficient improvement was achieved with BNSE at 500 mg kg^−1^, which outperformed 250 mg kg^−1^.

On the other hand, bile acid production is vital in the hepatic regulation of cholesterol homeostasis and cholesterol catabolism. Bilirubin is considered a physiologically important antioxidant [51], with beneficial effects at mildly elevated concentrations, and can neutralize ROS and prevent oxidative damage [52,53]. Recent studies have found that bilirubin helps to suppress immune reactions and enables prolonged tolerance to islet transplantation in diabetes [54]. These findings may potentially explain the increase in total bilirubin as a protective mechanism of the body against CYP free radicals. Interestingly, BNSE attenuated the T.B. levels to be consistent with those of the NR group, as similarly indicated by El-Dreny [55]. CYP reduces liver functioning and antioxidant enzymes while dramatically boosting liver enzyme levels, according to Golmohammadi et al. [45]. Our findings were consistent with Rajamurugan et al. [56], who discovered that a crude methanol extract of *B. nigra* leaf had no intrinsic toxicity and provided hepatoprotection against D-GalN-induced toxicity in rats. This could be due to its high phytochemical and bioactive compound concentrations [57]. As a result, BNSE can act as an antioxidant in acute liver injury. Interestingly, BNSE reduced the lipid peroxide levels to near-normal levels. It also lowered NO overproduction in rat hepatocytes, often connected to a lack of cytochrome P450 and damage [58].

### 3.5. Effects of B. nigra Sprout Extract on Kidney Function

Table 4 shows the nephroprotective effectiveness of BNSE at 250 and 500 mg kg^−1^ on the kidney function of CYP-induced immunosuppressed rats. CYP injection significantly increased (*p* < 0.05) the blood creatinine, urea, and BUN levels in the CYP group rats compared to the NR group, which is in line with the findings of Golmohammadi et al. [45]. High doses of BNSE significantly (*p* < 0.05) reduced the changes in creatinine, urea, and BUN produced by CYP injection. The creatinine, urea, and BUN levels were all reduced in a dose-dependent manner after receiving 250 and 500 mg kg^−1^ of BNSE. When the lower and higher doses were compared, creatinine was decreased by 18.31 and 35.92%, respectively. A similar pattern was seen in the urea level, with BNSE showing values of 31.18 and 51.25% at 250 and 500 mg kg^−1^, respectively. A more than 50% improvement in BUN was seen when rats were administered BNSE at 500 mg kg^−1^.

Indeed, CYP decreased renal performance and antioxidant enzyme activity, and it significantly increased (*p* < 0.05) the blood urea nitrogen–creatinine (BUN-Cr), malondialdehyde, nuclear factor kappa β (NF-kB), and interleukin 1 beta (IL-1B) concentrations in the study performed by Golmohammadi et al. [45]. Moreover, the obtained results were in line with Rajamurugan et al. [56], who indicated that a crude methanol extract of *B. nigra* leaf lacked inherent toxicity and exhibited hepatoprotective effects against D-GalN-induced toxicity in rats. This could be owing to its higher concentrations of phytochemicals and bioactive compounds, as evaluated by Tian and Deng. [57]. In the same year, Al-Qady and Shaban [59] confirmed the protective effect of a *B. nigra* seed extract against the physiological and histological effects of captopril on the rat kidney.

More clinical trials are required before the present work can be applied to humans, because this work was only conducted on experimental rats. Sprouts are a common culinary ingredient, but there is now sufficient evidence to support claims about their functional and physiological benefits. In addition, our previous research has shown that sprouting seeds increases their bioactive content and provides more health advantages than other methods [49,60,61,62,63]. However, additional laboratory and clinical trial research is necessary before any broad recommendations can be made based on the obtained findings.

## 4. Conclusions

Four experimental groups of rats were tested for BNSE’s hypolipidemic, hepatoprotective, and nephroprotective effects in cyclophosphamide-induced toxicity. The hydroalcoholic extraction of lab-grown *B. nigra* sprouts was carried out. BNSE at 500 mg kg^−1^ restored rats’ weights best after 3 weeks of oral therapy. In the hypolipidemic efficiency test, BNSE at 250 and 500 mg kg^−1^ significantly (*p* < 0.05) attenuated the TG, CHO, LDL-c, and VLDL-c levels in the CYP group. The atherogenicity was best improved when 500 mg kg^−1^ BNSE was applied compared to both the NR and CYP groups. The hepatoprotection and nephroprotection of BNSE led to improvements in liver and kidney function. The most effective amount of BNSE was 500 mg kg^−1^, surpassing 250 mg kg^−1^ in the CYP + BNSE250 group. Considering its hypolipidemic, hepatoprotective, and nephroprotective properties, BNSE may be helpful in disease treatment, but further clinical studies are recommended.

## Figures and Tables

**Table 1:** Weight gain % and organ weights of rats with CYP-induced immunosuppression when fed different doses of *B. nigra* sprout extract (mean ± SE), *n* = 8.

Item		Group *		
	NR	CYP	CYP + BNSE250	CYP + BNSE500
Initial BW (g)	177.61 ± 5.15	173.08 ± 4.27	179.12 ± 6.72	175.18 ± 5.01
Final BW (g)	207.56 ± 4.65	162.42 ± 4.18	187.28 ± 5.67	201.38 ± 4.21
BW gain %	16.86 ± 1.13 ^a^	−6.36 ± 1.21 ^d^	4.56 ± 1.07 ^c^	14.96 ± 2.11 ^b^
Liver weight %	4.78 ± 0.38 ^a^	3.73 ± 0.15 ^b^	4.86 ± 0.40 ^a^	5.20 ± 0.55 ^a^
Kidney weight %	0.83 ± 0.05 ^a^	0.77 ± 0.01 ^b^	0.79 ± 0.08 ^ab^	0.81 ± 0.03 ^ab^
Spleen weight %	0.29 ± 0.03 ^a^	0.25 ± 0.05 ^a^	0.30 ± 0.07 ^a^	0.34 ± 0.08 ^a^

SE: standard error; *: for experimental groups, see Materials and Methods; BW: body weight; ^a^,^b^,^c^ and ^d^: no significant difference (*p* > 0.05) between any two means within the same row with similar superscripted letters.

**Table 2:** Effects of *B. nigra* sprout extract at different doses on lipid profile parameters in rats with CYP-induced immunosuppression (mean ± SE), *n* = 8.

Group *	Lipid Profile Parameter (mg dL^−1^)				
	TG	CHO	HDL-C	LDL-C	VLDL-C
NR	82.53 ± 4.13 ^c^	102.52 ± 5.19 ^b^	48.16 ± 4.87 ^a^	37.85 ± 5.80 ^b^	16.51 ± 1.14 ^c^
CYP	149.34 ± 5.18 ^a^	161.15 ± 9.28 ^a^	33.19 ± 3.59 ^c^	98.09 ± 12.37 ^a^	29.87 ± 2.12 ^a^
CYP + BNSE250	97.19 ± 7.09 ^b^	95.28 ± 3.19 ^b^	42.19 ± 4.15 ^b^	33.69 ± 2.17 ^b^	19.43 ± 2.01 ^b^
CYP + BNSE500	80.49 ± 2.19 ^c^	96.14 ± 6.07 ^b^	46.08 ± 1.89 ^a^	33.96 ± 3.94 ^b^	16.10 ± 1.03 ^c^

*: for experimental groups, see Materials and Methods; TG: triglycerides; CHO: total cholesterol; HDL-C: high-density lipoprotein cholesterol; LDL-C: low-density lipoprotein cholesterol; VLDL-C: very low-density lipoprotein cholesterol; ^a^, ^b^ and ^c^: no significant difference (*p* > 0.05) between any two means within the same column with the same superscripted letters.

**Table 3:** Effects of *B. nigra* sprout extract at different doses on liver function in rats with CYP-induced immunosuppression (mean ± SE), *n* = 8.

Group *	**Liver Function**					
	**ALT (UL^−1^)**	**AST (UL^−1^)**		**ALP (UL^−1^)**		**T. Bili (mg dL^−1^)**
NR	76.27 ± 6.14 ^c^	102.54 ± 6.15 ^c^		85.19 ± 4.89 ^b^		0.72 ± 0.09 ^bc^
CYP	128.27 ± 12.73 ^a^	151.03 ± 17.25 ^a^		142.28 ± 8.51 ^a^		1.21 ± 0.19 ^a^
CYP + BNSE250	91.18 ± 7.98 ^b^	121.67 ± 9.17 ^b^		94.28 ± 9.17 ^b^		0.97 ± 0.12 ^ab^
CYP + BNSE500	85.48 ± 5.13 ^bc^	98.27 ± 8.27 ^c^		87.17 ± 8.11 ^b^		0.82 ± 0.08 ^bc^
Group *	**T.P. (g dL^−1^)**		**Albumin (g dL^−1^)**		**Globulin (g dL^−1^)**	
NR	10.02 ± 0.23 ^a^		5.71 ± 0.31 ^a^		4.31 ± 0.21 ^b^	
CYP	8.09 ± 0.23 ^c^		4.87 ± 0.09 ^c^		3.22 ± 0.18 ^c^	
CYP + BNSE250	9.24 ± 0.41 ^b^		5.11 ± 0.24 ^b^		4.13 ± 0.42 ^b^	
CYP + BNSE500	10.41 ± 0.32 ^a^		4.99 ± 0.47 ^b^		5.42 ± 0.35 ^a^	

*: for experimental groups, see Materials and Methods, ^a^, ^b^, and ^c^: no significant difference (*p* > 0.05) between any means within the same column with the same superscripted letters. ALT: alanine aminotransferase; AST: aspartate aminotransferase; ALP: alkaline phosphatase; T. Bili: total bilirubin.

**Table 4:** Effects of *B. nigra* sprout extract at different doses on kidney functions in rats with CYP-induced immunosuppression (mean ± SE), *n* = 8.

Group *	Kidney Function		
	Creatinine (mg dL^−1^)	Urea (mg dL^−1^)	BUN (mg dL^−1^)
NR	0.86 ± 0.09 ^c^	36.14 ± 3.58 ^c^	16.98 ± 4.28 ^c^
CYP	1.42 ± 0.13 ^a^	72.28 ± 5.24 ^a^	33.97 ± 3.55 ^a^
CYP + BNSE250	1.16 ± 0.45 ^b^	49.74 ± 3.89 ^b^	23.37 ± 3.01 ^b^
CYP + BNSE500	0.91 ± 0.11 ^bc^	35.24 ± 5.96 ^c^	16.58 ± 3.69 ^c^

*: for experimental groups, see Materials and Methods. BUN: blood urea nitrogen; ^a^, ^b^, and ^c^: no significant difference (*p* > 0.05) between any two means within the same column with the same superscripted letters.

## Data Availability

Data are contained within the article.
